# Workplace sexual harassment is associated with poor mental well-being among employees at a large Swedish university

**DOI:** 10.1080/16549716.2025.2465050

**Published:** 2025-02-19

**Authors:** Frida Pilgaard, Per-Olof Östergren, Gisela Priebe, Anette Agardh

**Affiliations:** a Division of Social Medicine and Global Health, Department of Clinical Sciences, Malmö, Lund University, Malmö, Sweden; b Department of Social and Psychological Studies, Karlstad University, Karlstad, Sweden

**Keywords:** Sexual harassment, academia, mental health, vitality, mental well-being

## Abstract

**Background:**

Sexual harassment (SH) is a persistent problem at workplaces around the world, including academia.

**Objective:**

This study examines the association between SH and mental well-being among employees at Lund University (LU) in Sweden.

**Methods:**

Data was obtained from a cross-sectional survey targeting all LU employees in 2019. SH exposure was measured using a ten-item scale capturing SH experiences and enabling the differentiation between soliciting and non-soliciting types of SH. Validated instruments were used to measure two aspects of mental well-being; mental health (GHQ-12) and vitality (SF-36 vitality scale). Association between SH exposure and outcome variables was investigated through multivariable logistic regression analysis adjusting for confounders. Modifying effects of gender, age, background and academic position, respectively, on the relation between SH and outcomes were studied.

**Results:**

Workplace SH was associated with poor mental health (PMH) (OR 1.5 (95% CI 1.1–2.0)) and low vitality (LV) (OR 1.8 (95% CI 1.3–2.5)) among women and with LV (OR 2.0 (95% CI 1.1–3.9)) among men, after adjusting for confounders. Among women, experiences of non-soliciting and soliciting SH combined were associated with PMH and LV. Among men, experiences of non-soliciting SH behaviours exclusively were associated with LV. Indications of synergistic interaction affecting the association between SH and LV were found related to age, background and academic position, but not gender.

**Conclusions:**

Workplace SH is a significant risk factor for poor mental well-being, primarily among female, but also among male university employees. These findings can inform local policies for prevention of SH.

## Paper context

- **Main findings:** The main findings of this article concern negative consequences of sexual harassment on mental well-being among employees at a large Swedish university, which broadens the existing knowledge base to a Scandinavian setting.
- **Added knowledge:** The study adds new knowledge regarding association between workplace sexual harassment and vitality, different types of sexual harassment and their relation to mental well-being as well as the modifying effects of gender, age, background and academic position on the relation between sexual harassment and mental well-being.
- **Global health impact for policy and action:** These findings can inform local policies for prevention of SH.

## Background

Sexual harassment (SH) is a persistent problem at workplaces around the world [1,2]. In a recent EU-wide survey, about one in five women reported experiences of SH within the past twelve months, with women in employment reporting these types of experiences more often compared to those who had never done paid work [3]. Academia is no exception, and at Lund University in Sweden, the largest university in Sweden in terms of number of employees, 25% of all female and 7% of all male employees report experiences of SH in connection with their work [4,5]. The perpetrator of SH is often a man and the target a woman, even if other situations also are reported such as SH by men towards other men and SH by women towards men or other women [6]. Further, the target often belongs to a vulnerable group, for example, persons of young age, having insecure employment conditions or belonging to an ethnic or sexual minority [1]. Higher levels of SH are also found among women not meeting traditional feminine ideals by having characteristics considered more masculine or having positions of leadership at the workplace [3,7,8]. Organisational factors such as tolerance for SH and the job gender balance have also been shown to play a role in the occurrence of SH at a workplace [9].

It is well demonstrated in previous research that SH at the workplace leads to multiple negative health- and work-related outcomes such as anxiety, depression, general stress, decreased job satisfaction and decreased organisational commitment [9–11]. Recent studies from Scandinavia show increased risk of depressive symptoms, psychotropic medication, long-term sickness absence and suicidal behaviour after SH exposure [12–16]. Outcomes related to the victims’ mental well-being are the most commonly studied health consequences of SH [9,11]. However, few, if any, studies have examined the relation between SH and vitality. Vitality, referring to the individual’s experience of energy and fatigue, has been found to be positively related to employees perceived effective functioning, sustainable employability, participation as well as creativity and innovative behaviours, and negatively related to societal costs [17–22]. Also, few studies examine the relation between different types of sexual harassment and mental well-being outcomes. In a meta-analysis by Sojo et al, a broad differentiation is made between different types of SH by intensity, categorising experiences such as sexual coercion and unwanted sexual attention as being intense and experiences of sexist organisational climate and gender harassment as less intense [23]. The results indicated that more intense yet less frequent harmful experiences and less intense but more frequent harmful experiences had similar negative effects on women’s well-being [23]. This meta-analysis included women only, and to our knowledge, studies examining different types of SH and their consequences among men are scarce.

Sexually harassed women generally report worse outcomes compared to sexually harassed men. However, findings regarding gender differences in consequences of SH are mixed, and few studies specifically explore SH among men [2,11]. In a meta-analytic review examining psychological outcomes of workplace SH, Chan and colleagues found no stronger impact of SH on female compared to male employees [24]. Despite mixed findings regarding gender differences in the consequences of SH, the research evidence suggests women face greater harm from SH as compared to men because they are far more likely to be exposed to SH [11]. Apart from gender, there are research findings indicating that the power relation between the target and the harasser, perceived organisational support and age are factors modifying the harm of SH [11,24]. Minority ethnicity status of the target has also been suggested to exacerbate the harm of SH, although more research is needed specifically comparing outcomes for minority and non-minority women [11].

A majority of studies focusing on SH in academia use empirical data from USA or other English speaking countries, and SH research from non-English speaking countries is needed in order to broaden the picture [5]. There is currently no ‘gold standard’ regarding methods for measuring levels of SH at workplaces, which limits comparability across SH studies [3]. Another factor reducing comparability between studies is differences in definitions used. For example, in the USA gender harassment is often considered a form of sexual harassment, which is not the case in Sweden where those concepts are separated [25,26]. Further, perceptions of what constitutes SH vary between individuals and across cultures. For example, in Europe higher prevalence of SH is reported in countries that score high on the gender equality index, such as Sweden, compared to countries that score low [27]. This is explained by greater awareness of SH in these countries and of it being an abuse of fundamental rights, leading to a greater willingness to report experiences of SH in surveys [27]. The cultural relativeness surrounding what constitutes SH and how it is defined make it relevant to examine exposure to workplace SH and its relation to individuals’ mental well-being in a Swedish academic context. Consequently, the aim of this study was to examine the association between SH and mental well-being, including vitality, among employees at a large Swedish university. Associations were examined separately by gender to identify possible gender differences. A secondary aim was to examine the modifying effects of gender, age, background and academic position on the relation between SH and mental well-being. We hypothesised that poorer mental well-being outcomes related to SH would be found among women, young employees and employees with a foreign background or low academic position.

## Methods

### Data collection

This study is part of a larger project named ‘Tellus’, initiated at Lund University (LU), Sweden, 2018, with the aim to strengthen preventive work against sexual harassment (SH) at the university [28]. As part of the Tellus project, a cross-sectional survey was conducted at LU in 2019 targeting both staff/PhD students and students. The survey included questions related to experiences of SH as well as mental health status and vitality. The overall results from the survey and details about the survey instrument have been published elsewhere [4,29].

### Study population

A survey, in both English and Swedish, was sent out by email to all employees at LU (*N* = 8,238) in November 2019. The response rate was 33% (*n* = 2,750). After exclusion of those with missing data on both sex and gender and those who did not answer any of the 10 questions on experiences of SH, altogether 14 individuals, the final study population consisted of 2,736 individuals.

When comparing the participants and the target population by means of employment records, no major differences were found apart from a few observations. Women were slightly over-represented among study participants and both men and women participants tended to be somewhat older compared to the target group. Also, a slight over-representation of employees with permanent employment (versus temporary) was seen [4].

### Exposure variables sexual harassment

The instrument used to measure SH in this study was the ‘Lund University Sexual Harassment Inventory’ (LUSHI), developed and validated within the Tellus project [29]. LUSHI is an instrument based on previous research concerning instruments used for assessing sexual harassment, as well as a large number of individual interviews and focus group discussions that informed the decisions concerning the selection of what items to include in the scale. The reliability and construct validity of the instrument have been demonstrated in a previous study showing a Cronbach´s alpha of 0.80 [29]. In the same study, two factors were identified considered to cover two central, but different, aspects of SH, measurable by two subscales; unwanted sexual attention of non-soliciting type and unwanted sexual attention of soliciting type. The first factor, non-soliciting SH, is considered to represent a more general climate of SH at a certain workplace, including items such as unwelcome suggestive looks or gestures, while the second factor, soliciting SH, is considered to represent a more explicit effort to create a sexual relation between the perpetrator and the victim, including items such as unwelcome soliciting or pressuring for ‘dates’ [29].

The full scale consists of the following list of ten situations/behaviours, including one situation representing sexual violence: 1. Unwelcome suggestive looks or gestures, 2. Unwelcome ‘inadvertent’ brushing or touching, 3. Unwelcome bodily contact such as grabbing or fondling, 4. Unwelcome comments, 5. Unwelcome soliciting or pressuring for ‘dates’, 6. Unwelcome gifts, 7. Unwelcome contact by post or telephone, 8. Unwelcome contact online, for example, by social media or email, 9. Stalking, and 10. Attempts to conduct or the conduct of oral, vaginal or anal sex or other equivalent sexual activity in which one did not participate voluntarily. The first four scale items listed above were included in the non-soliciting subscale, while scale items five through nine were included in the soliciting scale. Item 10, representing sexual violence, fell outside the two factors emerging in the factor analysis and was consequently excluded from the two subscales [29]. It was specified in the instructions to the respondents that the situations should have a clear sexual connotation. The study participants were asked if they had experienced any of these situations in connection with their employment at LU with the answer options: Yes, once; Yes, more than once; and No.

To create our main exposure variable, SH during the last three years at LU, all participants who answered yes to at least one of the 10 situations listed in the full scale, and who indicated that the situation/s occurred in the last 12 months or between one and three years ago, were classified as exposed to SH, and all others as non-exposed.

To examine SH experiences by non-soliciting or soliciting type, an additional exposure variable, SH by type, was created, using the same time frame as the main exposure variable (situation/s occurred in the last 12 months or between one and three years ago). Participants who answered yes to at least one of the items included in the non-soliciting scale and no to all items in the soliciting scale were classified as exposed to non-soliciting SH exclusively, participants who answered yes to at least one of the items included in the soliciting scale and no to all items in the non-soliciting scale were classified as exposed to soliciting SH exclusively, participants who answered yes to at least one of the items included in the non-soliciting scale as well as yes to at least one item in the soliciting scale were classified as exposed to non-soliciting and soliciting SH combined, and all other as non-exposed to non-soliciting or soliciting SH during the last three years at LU.

### Outcome variables mental health and vitality

Two outcome variables, reflecting mental well-being, were used in this study: mental health and vitality. To assess the current mental health status of the participants, we used the 12-item version of the General Health Questionnaire (GHQ-12), a widely recognised screening instrument for assessing psychological distress in individuals [30]. The instrument consists of 12 questions, with response options on a four-point Likert scale capturing symptoms experienced during the past few weeks. The responses were dichotomised in line with the standard scoring method (0-0-1-1) generating a total score ranging from 0–12 per individual. The mean score was 2.15, and therefore, the threshold for defining a case was set at ≥3 in line with existing recommendations [31]. To assess the participants’ vitality, we used the vitality scale of the Short-Form Health Survey (SF-36) questionnaire [19]. The scale consists of four items capturing experiences of energy level and fatigue during the past four weeks and includes questions like ‘How much of the time during the past four weeks have you felt worn out?’. Four response options were given on a four-point Likert scale. Each item was scored on a 0 to 100 range, in line with recommendations, with the scoring values 0, 33, 66, or 100 [32]. The item score values were then added and divided by the number of items to create a scale score for each individual. The threshold for low vitality was set at the population tertile level, which was ≤40 for employees.

In the following sections of this article, we use mental well-being as an umbrella term when referring collectively to the two outcomes mental health and vitality.

### Background variables

Two gender-related questions were used in the survey; ‘what gender were you assigned at birth?’ (female/male) and ‘what is your current gender identity?’ (Female/male/I do not identify as male or female). The answer to the second question was used to categorise participants as women, men or nonbinary. However, for participants with missing information on this question (*n* = 15), the answer to the first question was used.

Information about age was obtained by asking the study participants to which of the following age groups they belonged: 30 years or younger, 31–40 years, 41–49 years, 50–59 years or 60 years or older.

Participants who were either born abroad or had two parents born abroad were categorised as individuals with a foreign background, in line with the definition used by Statistics Sweden [33]. Participants lacking information about parents’ country of birth were presumed to have Swedish background if they were born in Sweden (*n* = 5).

Professional position was specified according to nine types in the survey which were aggregated into the following six categories for the purpose of analysis: ‘professors’, ‘senior lecturer’, ‘lecturer and researcher’, ‘PhD students’, ‘administrative and technical support staff’, and ‘others’.

Further, a variable was created dichotomising the participants into a ‘high’ vs. ‘low or other’ academic position, based on their professional position. Participants were categorised as having a ‘high’ academic position if they had the professional position ‘professor’ or ‘senior lecturer’, and the remaining professional positions were categorised together in one group as a ‘low or other’ academic position.

Participants were also asked whether their form of employment was permanent or temporary.

### Statistical analysis

We investigated the associations between background variables as well as exposure variables and the mental well-being outcomes through bivariate logistic regression analysis. To further investigate the association between our exposure variables and mental well-being outcomes we used multivariable logistic regression analysis. We included two different models adjusting for stepwise added confounders (age, background (Swedish/foreign), type of employment and professional position). The first model included age only, as this was considered to be the most important confounder candidate. In the second model the variables background (Swedish/foreign), type of employment and professional position were added to obtain the fully adjusted estimates. The selection of confounders was guided by previous research identifying factors associated with our exposure variable, as outlined in the background section, and also recognised as related to mental well-being. The results are presented as odds ratios and 95% CI. Additive interaction analyses were calculated as proposed by Rothman according to which a synergy index >1 indicates a synergistic effect and a synergy index <1 an antagonistic effect [34]. Unadjusted ORs were calculated for dummy variables combining gender with SH, age (≤40 years vs 40+ years) with SH, background (foreign vs Swedish) with SH and academic position (‘high’ vs ‘low or other’) with SH. As a sensitivity analysis, the same calculations were made using ORs adjusted for age.

Data from nonbinary participants are often excluded in descriptive statistics due to small numbers. In this study, we included them in the descriptive analyses in order to make our data available for future pooled studies. However, due to small numbers, we excluded nonbinary participants (24 out of 2,736) from further analysis.

Missing values were treated as missing and reported for each variable in result tables. All analyses were made separately for men and women. Statistical analyses were performed in Stata version 17.

### Ethical considerations

The study was approved by the Regional Ethical Review Board in Lund (Dnr 2018/350) and was in accordance with the 1964 helsinki declaration and its later amendments or comparable ethical standards.

## Results

In total, 2,736 individuals participated in the study (57% women, 42% men and 0.9% of nonbinary gender). No major differences were found between women and men regarding background variables, apart from men generally reporting higher professional positions compared to women. Table 1 shows the characteristics of the sample including background variables, prevalence of SH, poor mental health (PMH) and low vitality (LV).

**Table 1.** Characteristics of the sample, prevalence of sexual harassment (SH), poor mental health (PMH) and low vitality (LV), separately by gender.

		Women *n* = 1,551		Men *n* = 1,161		Nonbinary *n* = 24	
		n	%	n	%	n	%
Age group							
	≤30	188	12.1	144	12.4	3	12.5
	31–40	373	24.1	250	21.5	11	45.8
	41–49	467	30.1	300	25.8	5	20.8
	50–59	365	23.5	320	27.6	2	8.3
	≥60	158	10.2	147	12.7	3	12.5
	Missing	0	0	0	0	0	0
Professional position							
	Professors	81	5.2	203	17.5	2	8.3
	Senior Lecturers	189	12.2	193	16.6	3	12.5
	Lecturers and Researchers	243	15.7	227	19.6	5	20.8
	PhD Student	223	14.4	170	14.6	5	20.8
	Administrative & Technical	758	48.9	334	28.8	7	29.2
	Other	56	3.6	33	2.8	2	8.3
	Missing	1	0.1	1	0.1	0	0
Type of employment							
	Permanent	1,111	71.6	817	70.4	15	62.5
	Temporary	420	27.1	314	27.1	9	37.5
	Missing	20	1.3	30	2.6	0	0
Background							
	Swedish	1,183	76.3	868	74.8	15	62.5
	Foreign	368	23.7	291	25.1	9	37.5
	Missing	0	0	2	0.2	0	0
SH at LU last three years		216	13.9	55	4.7	5	20.8
	Missing	0	0	0	0	0	0
SH by type							
	Non-soliciting exclusively	116	7.5	32	2.8	3	12.5
	Soliciting exclusively	22	1.4	12	1.0	0	0
	Non-soliciting and soliciting combined	78	5.0	11	1.0	2	8.3
	Missing	0	0	0	0	0	0
PMH		517	33.3	267	23.0	12	50.0
	Missing	19	1.2	23	2.0	0	0
LV		451	29.1	217	18.7	8	33.3
	Missing	63	4.1	60	5.2	2	8.3

Association between background variables, sexual harassment and mental well-being in the bivariate logistic regression analysis.

### Prevalence of sexual harassment, poor mental health and low vitality

Women reported higher prevalence of PMH, LV and experiences of SH compared to men. Among women, 33% were categorised as having PMH, 29% LV and 14% reported experiences of SH in connection with work during the last three years. Among men, 23% were categorised as having PMH, 19% LV and 5% reported experiences of SH in connection with work during the last three years (Table 1). Regarding type of SH, the most common form reported was non-soliciting SH exclusively in all gender groups. Among women, it was also relatively common to have experienced non-soliciting and soliciting types of SH combined (5%) while few reported experiences of soliciting SH exclusively (1%). Among men, few reported experiences of non-soliciting only (1%) or non-soliciting and soliciting combined (1%). Highest prevalence of PMH, LV and SH experiences was found in the nonbinary group (Table 1).

Associations between background variables, SH and mental well-being outcomes are presented in Table 2 for women and men, respectively. Among women, experiences of SH during the last three years at LU were statistically significantly associated with PMH (OR 1.6 (95%- CI 1.2–2.2)) and LV (OR 1.9 (95% CI 1.4–2.6)). Among men, elevated odds ratios were seen for both outcomes although not statistically significant; OR 1.3 (95%- CI 0.7–2.3) for PMH and OR 1.7 (95% CI 0.9–3.2) for LV.

**Table 2.** Bivariate logistic regression analysis showing the association between sexual harassment (SH) at Lund University (LU), background variables and poor mental health (PMH) and low vitality (LV) among women and men, respectively. Missing data reported in parentheses.

		PMH OR (95% CI)		LV OR (95% CI)	
Independent variables		Women (*n* = 1,532)	Men (*n* = 1,138)	Women (*n* = 1,488)	Men (*n* = 1,101)
SH at LU last three years					
	Not exposed	1.0 (ref)	1.0 (ref)	1.0 (ref)	1.0 (ref)
	Exposed	1.6 (1.2–2.2)	1.3 (0.7–2.3)	1.9 (1.4–2.6)	1.7 (0.9–3.2)
SH by type					
	Not exposed	1.0 (ref)	1.0 (ref)	1.0 (ref)	1.0 (ref)
	Non-soliciting SH exclusively	1.2 (0.8–1.8)	1.8 (0.8–3.7)	1.6 (1.1–2.4)	2.3 (1.1–4.9)
	Soliciting SH exclusively	1.8 (0.8–4.2)	0.7 (0.1–3.1)	1.9 (0.8–4.4)	0.9 (0.2–4.1)
	Non-soliciting and soliciting SH combined	2.5 (1.6–4.0)	0.8 (0.2–3.5)	2.7 (1.7–4.3)	1.0 (0.2–4.6)
Age group					
	≤30	7.6 (4.4–13.1)	6.7 (3.3–13.5)	3.6 (2.1–6.2)	2.6 (1.3–5.2)
	31–40	4.5 (2.7–7.4)	6.4 (3.3–12.4)	3.1 (1.9–5.1)	3.9 (2.1–7.2)
	41–49	2.9 (1.8–4.8)	3.6 (1.8–7.0)	2.5 (1.5–4.0)	2.1 (1.1–4.0)
	50–59	2.3 (1.4–3.9)	2.7 (1.4–5.3)	1.7 (1.04–2.9)	1.6 (0.9–3.1)
	≥60	1.0 (ref)	1.0 (ref)	1.0 (ref)	1.0 (ref)
Professional position (missing: women *n*=1, men *n*=1)					
	Professor	1.0 (ref)	1.0 (ref)	1.0 (ref)	1.0 (ref)
	Senior Lecturers	1.8 (0.9–3.3)	1.5 (0.8–2.7)	0.8 (0.5–1.6)	1.9 (1.03–3.4)
	Lecturers and Researchers	2.2 (1.2–4.0)	3.5 (2.1–5.9)	1.1 (0.6–2.0)	3.0 (1.7–5.2)
	PhD Student	3.9 (2.1–7.2)	5.1 (3.0–8.7)	1.9 (1.1–3.4)	3.0 (1.7–5.4)
	Administrative & Technical	1.7 (0.98–3.1)	2.1 (1.3–3.5)	1.2 (0.7–2.0)	2.1 (1.2–3.6)
	Other	1.9 (0.9–4.2)	2.2 (0.8–5.6)	0.8 (0.3–1.8)	0.9 (0.3–3.3)
Type of employment (missing: women *n*=20, men *n*=30)					
	Permanent	1.0 (ref)	1.0 (ref)	1.0 (ref)	1.0 (ref)
	Temporary	2.0 (1.6–2.6)	2.6 (1.9–3.4)	1.5 (1.2–1.9)	1.7 (1.2–2.4)
Background (missing: men *n*=2)					
	Swedish	1.0 (ref)	1.0 (ref)	1.0 (ref)	1.0 (ref)
	Foreign	1.2 (0.96–1.6)	1.6 (1.2–2.1)	1.0 (0.8–1.3)	1.4 (0.98–1.9)

Regarding type of SH, experiences of non-soliciting and soliciting SH combined were statistically significantly associated with both outcomes among women; OR 2.5 (95% CI 1.6–4.0) for PMH and OR 2.7 (95% CI 1.7–4.3) for LV. Non-soliciting SH exclusively was statistically significantly associated with LV among both women and men; OR 1.6 (95% CI 1.1–2.4) among women and OR 2.3 (95% CI 1.1–4.9) among men.

The results for women show statistically significantly higher odds ratios for PMH in the younger age groups compared to the oldest, among PhD students and lecturers and researchers compared to professors and among employees with temporary employment form compared to employees with permanent employment form. The same pattern was seen for LV except that elevated odds ratios for LV among lecturers and researchers were not statistically significant.

Among men, statistically significantly higher odds ratios for PMH were seen among younger age groups compared to older, among lecturers and researchers, PhD students and administrative & technical staff compared to professors, among employees with temporary employment form compared to permanent employment form and among study participants with foreign background compared to study participants with Swedish background. The same pattern was seen for LV except that elevated odds ratios for LV among study participants with foreign background were not statistically significant.

### Association between sexual harassment and mental well-being after adjusting for confounders

The association between experience of SH and PMH and LV among women remained significant after adjusting for age, background, type of employment and professional position, with an OR of 1.5 (95% CI 1.1–2.0) for PMH and OR 1.8 (95% CI 1.3–2.5) for LV (Table 3). Among men, experience of SH was not associated with any of the mental well-being outcomes in the bivariate logistic regression model (Table 2). However, a significant association was found with LV (OR 2.0 (95% CI 1.1–3.9)) after adjusting for age, background, type of employment and professional position (Table 3).

**Table 3.** Association between exposure to sexual harassment (SH) at Lund University (LU) during the last three years and poor mental health (PMH) and low vitality (LV).

	Model 1* PMH		Model 2** PMH		Model 1* LV		Model 2** LV	
Independent variables	Women	Men	Women	Men	Women	Men	Women	Men
SH at LU last three years								
Not exposed	1.0 (ref)	1.0 (ref)	1.0 (ref)	1.0 (ref)	1.0 (ref)	1.0 (ref)	1.0 (ref)	1.0 (ref)
Exposed	1.4 (1.1–1.9)	1.4 (0.7–2.7)	1.5 (1.1–2.0)	1.6 (0.8–3.0)	1.8 (1.3–2.4)	1.8 (0.99–3.5)	1.8 (1.3–2.5)	2.0 (1.1–3.9)
Age group								
≤30	7.4 (4.3–12.7)	6.8 (3.2–13.7)	5.9 (3.2–11.1)	2.9 (1.2–6.8)	3.4 (2.0–5.8)	2.7 (1.4–5.3)	2.8 (1.5–5.2)	1.8 (0.8–4.1)
31–40	4.3 (2.6–7.2)	6.5 (3.3–12.7)	4.0 (2.3–6.9)	3.7 (1.7–8.0)	2.9 (1.8–4.8)	4.0 (2.2–7.5)	2.7 (1.6–4.5)	2.7 (1.3–5.6)
41–49	2.9 (1.7–4.7)	3.6 (1.8–7.1)	2.9 (1.7–4.8)	3.2 (1.5–6.6)	2.4 (1.5–3.9)	2.2 (1.2–4.1)	2.3 (1.4–3.8)	1.7 (0.9–3.4)
50–59	2.3 (1.4–3.9)	2.8 (1.4–5.4)	2.4 (1.4–4.1)	2.6 (1.3–5.3)	1.7 (0.9–3.2)	1.7 (0.9–3.2)	1.7 (0.99–2.8)	1.5 (0.8–2.9)
≥60	1.0 (ref)	1.0 (ref)	1.0 (ref)	1.0 (ref)	1.0 (ref)	1.0 (ref)	1.0 (ref)	1.0 (ref)
Background								
Swedish			1.0 (ref)	1.0 (ref)			1.0 (ref)	1.0 (ref)
Foreign			1.0 (0.7–1.3)	1.2 (0.8–1.6)			0.9 (0.7–1.1)	1.1 (0.8–1.6)
Professional position								
Professors			1.0 (ref)	1.0 (ref)			1.0 (ref)	1.0 (ref)
Senior Lecturers			1.4 (0.7–2.7)	1.1 (0.6–1.9)			0.6 (0.3–1.2)	1.6 (0.8–3.0)
Lecturers and Researchers			1.3 (0.6–2.5)	1.8 (1.01–3.3)			0.7 (0.4–1.4)	1.9 (0.99–3.0)
PhD Student			1.4 (0.7–3.0)	2.2 (1.1–4.6)			1.0 (0.5–2.1)	1.7 (0.8–3.8)
Administrative & Technical			1.2 (0.7–2.3)	1.4 (0.8–2.4)			0.8 (0.5–1.4)	1.6 (0.9–3.0)
Other			1.5 (0.6–3.4)	1.0 (0.4–3.1)			0.7 (0.3–1.5)	0.8 (0.2–3.2)
Type of employment								
Permanent			1.0 (ref)	1.0 (ref)			1.0 (ref)	1.0 (ref)
Temporary			1.2 (0.8–1.8)	1.4 (0.9–2.3)			1.1 (0.7–1.6)	1.2 (0.7–1.9)

*Model 1: Adjusted for age. Number of observations included in PMH model 1; women = 1,532, men =1,138. Number included in LV model 1; women=1,488, men =1,101.

**Model 2: Adjusted for age, background, professional position and type of employment. Number of observations included in PMH model 2; women = 1,511, men = 1,110. Number included in LV model 2; women=1,468, men =1,172.

Odds ratios and 95% confidence intervals presented for two different multivariable logistic regression models by gender.

Regarding different types of SH, among women, experiences of non-soliciting and soliciting SH combined were statistically significantly associated with both outcomes after adjusting for age, background, type of employment and professional position; OR 2.5 (95% CI 1.5–4.1) for PMH and OR 2.6 (95% CI 1.7–4.2) for LV (Table 4). The association between experiences of non-soliciting SH exclusively and LV found in this group in the bivariate logistic regression analysis (Table 2) did not remain statistically significant after adjusting for confounders. Among men, experiences of non-soliciting SH exclusively were statistically significantly associated with LV after adjusting for confounders, yielding an OR of 2.5 (95% CI 1.2–5.5) (Table 4).

**Table 4.** Association between exposure to different types of sexual harassment (SH), non-soliciting, soliciting or both types, at Lund University (LU) during the last three years and poor mental health (PMH) and low vitality (LV). Odds ratios and 95% confidence intervals presented for two different multivariable logistic regression models by gender.

Independent variables	Model 1* PMH		Model 2** PMH		Model 1* LV		Model 2** LV	
	Women	Men	Women	Men	Women	Men	Women	Men
SH at LU last three years by type								
Not exposed non-soliciting/soliciting SH	1.0 (ref)	1.0 (ref)	1.0 (ref)	1.0 (ref)	1.0 (ref)	1.0 (ref)	1.0 (ref)	1.0 (ref)
Exposed Non-soliciting SH exclusively	1.0 (0.7–1.5)	1.7 (0.8–3.6)	1.0 (0.7–1.5)	1.7 (0.8–3.7)	1.4 (0.9-2-1)	2.5 (1.1–5.3)	1.4 (0.95–2.1)	2.5 (1.2–5.5)
Exposed Soliciting SH exclusively	1.7 (0.7–4.1)	1.1 (0.2–5.7)	1.7 (0.7–4.2)	1.4 (0.3–7.7)	1.8 (0.8–4.5)	1.2 (0.2–5.3)	1.9 (0.8–4.7)	1.5 (0.3–7.6)
Exposed Non-soliciting and soliciting SH combined	2.3 (1.4–3.7)	0.9 (0.2–4.4)	2.5 (1.5–4.1)	1.2 (0.2–5.9)	2.4 (1.5–3.9)	1.1 (0.2–5.3)	2.6 (1.7–4.2)	1.3 (0.3–6.3)
Age group								
≤30	7.5 (4.4–13.1)	6.6 (3.3–13.5)	6.1 (3.2–11.4)	2.9 (1.2–6.8)	3.5 (2.0–5.9)	2.6 (1.3–5.2)	2.8 (1.5–5.2)	1.7 (0.7–4.1)
31–40	4.5 (2.7–7.5)	6.4 (3.3–12.5)	4.1 (2.4–7.1)	3.7 (1.7–7.9)	3.0 (1.8–4.9)	3.9 (2.1–7.3)	2.7 (1.6–4.6)	2.7 (1.3–5.5)
41–49	2.9 (1.8–4.8)	3.6 (1.8–7.0)	2.9 (1.7–4.9)	3.2 (1.5–6.5)	2.4 (1.5–4.0)	2.1 (1.1–4.0)	2.3 (1.4–3.9)	1.7 (0.8–3.3)
50–59	2.4 (1.4–4.0)	2.7 (1.4–5.4)	2.5 (1.5–4.2)	2.6 (1.3–5.3)	1.8 (1.1–2.9)	1.7 (0.9–3.1)	1.7 (0.99–2.8)	1.5 (0.8–2.9)
≥60	1.0 (ref)	1.0 (ref)	1.0 (ref)	1.0 (ref)	1.0 (ref)	1.0 (ref)	1.0 (ref)	1.0 (ref)
Background								
Swedish			1.0 (ref)	1.0 (ref)			1.0 (ref)	1.0 (ref)
Foreign			1.0 (0.7–1.3)	1.2 (0.8–1.6)			0.9 (0.7–1.1)	1.1 (0.8–1.6)
Professional position								
Professors			1.0 (ref)	1.0 (ref)			1.0 (ref)	1.0 (ref)
Senior Lecturers			1.4 (0.7–2.7)	1.1 (0.6–2.0)			0.6 (0.3–1.1)	1.6 (0.8–3.1)
Lecturers and Researchers			1.3 (0.7–2.6)	1.8 (1.01–3.4)			0.7 (0.4–1.4)	1.9 (0.99–3.7)
PhD Student			1.5 (0.7–3.2)	2.2 (1.1–4.7)			1.1 (0.5–2.2)	1.7 (0.8–3.8)
Administrative & Technical			1.3 (0.7–2.3)	1.4 (0.8–2.4)			0.8 (0.5–1.5)	1.7 (0.9–3.0)
Other			1.6 (0.7–3.7)	1.0 (0.4–3.1)			0.7 (0.3–1.6)	0.8 (0.2–3.2)
Type of employment								
Permanent			1.0 (ref)	1.0 (ref)			1.0 (ref)	1.0 (ref)
Temporary			1.2 (0.8–1.7)	1.4 (0.9–2.3)			1.0 (0.7–1.5)	1.2 (0.7–1.9)

*Model 1: Adjusted for age. Number of observations included in PMH model 1; women = 1,532, men =1,138. Number included LV model 1; women=1,488, men =1,101.

**Model 2: Adjusted for age, background, professional position and type of employment. Number of observations included in PMH model 2; women = 1,511, men = 1,110. Number included LV model 2; women=1,468, men =1,172.

### Interaction analyses

Combining SH exposure with gender, we found indication of synergistic interaction, with women exposed to SH seemingly more vulnerable for LV and PMH. However, this result did not remain in the sensitivity analyses using ORs adjusted for age (data not shown). No indications of interaction between SH exposure and any of the studied variables age, academic position and foreign background influencing the probability of PMH remained after sensitivity analyses using ORs adjusted for age (data not shown).

We found indication of synergistic interaction between exposure to SH and age (only among men), background and academic position regarding the probability of LV. Among women, having a foreign background seemed to increase the vulnerability for LV (SI 2.6) (Table 5). Among men, having a young age seemed to increase the vulnerability for LV (SI 10.0) and having a foreign background seemed to mitigate this potential effect (SI 0.3). However, low numbers of double exposed (nine or less) in the two different dummy variables make this result uncertain (Table 5). Having a ‘low or other’ academic position seemed to increase the vulnerability for LV among both women (SI 2.1) and men (SI 5.5) (Table 5). We performed the tests without adjustment for confounders, but the afore mentioned results were stable in the sensitivity analysis using ORs adjusted for age.

**Table 5.** Interaction analyses result, performed by using dummy variables combining age, background and academic position, respectively, with sexual harassment (SH) exposure during the last three years. Unadjusted ORs with 95% CI and synergy index SI presented for low vitality, by gender.

	Women			Men		
Dummy variables	n	OR (CI 95%)	SI	n	OR (CI 95%)	SI
**Age + SH exposure (≤ 40 years vs 40+ years)**						
Old + no SH	827	Ref category		689	Ref category	
Old + SH	107	1.8 (1.2–2.8)		38	1.0 (0.4–2.4)	
Young + no SH	440	1.6 (1.3–2.1)		360	1.9 (1.4–2.5)	
Young + SH	104	2.9 (1.9–4.4)		14	9.6 (3.2–29.1)	
			1.3			10.0
**Background + SH exposure**						
Swedish + no SH	979	Ref category		789	Ref category	
Swedish + SH	156	1.7 (1.2–2.4)		39	2.0 (1.01–4.1)	
Foreign + no SH	298	0.9 (0.7–1.2)		259	1.4 (1.01–2.0)	
Foreign + SH	55	2.6 (1.5–4.5)		13	1.4 (0.4–5.1)	
			2.6			0.3
**Academic position + SH exposure (“high” vs “low or other”)**						
“High” + no SH	206	Ref category		347	Ref category	
“High” + SH	48	1.5 (0.8–3.1)		23	0.9 (0.3–3.2)	
“Low” or other + no SH	1,070	1.4 (0.98–2.0)		701	1.7 (1.2–2.4)	
“Low” or other + SH	163	3.0 (1.9–4.6)		29	4.3 (2.0–9.5)	
			2.1			5.5

## Discussion

This study contributes with new knowledge regarding SH exposure and its association with mental well-being outcomes, including vitality, among employees in a Scandinavian academic workplace setting. We found that experiences of SH during the last three years at the workplace were statistically significantly associated with PMH and LV among female employees, after adjusting for age, background, type of employment and professional position. Among men, experiences of SH were statistically significantly associated with LV after adjusting for age, background, type of employment and professional position. More specifically, among women, experiences of non-soliciting and soliciting SH combined were associated with both PMH and LV. Among men, the only type of SH that was statistically significantly associated with any mental well-being outcome was experiences of non-soliciting SH associated with LV. These results indicate that SH exposure is a significant risk factor for poor mental well-being, primarily among female, but also among male university employees.

Our result is in line with previous studies showing workplace SH being negatively related to women’s mental well-being [23]. Previous findings suggest that SH taking place over a longer period of time and more frequent experiences cause poorer psychological outcomes [1,35]. This could partly explain our finding that experiences of non-soliciting and soliciting SH combined had the strongest association with poor mental well-being among women in our study, as experiences of a combination of different types of SH can be an indicator of more frequent experiences of SH or experiences taking place over a longer period of time.

Previous studies regarding men’s reactions to SH experiences show more mixed results. Some studies indicate that male targets of SH perceive these experiences to be less anxiety provoking, bothersome or stressful compared to women, while other studies indicate negative consequences of SH for men’s mental well-being, similar to the consequences for women [1,11]. We believe our study provides valuable insight regarding gender differences related to different types of SH and poor mental well-being. In our study, we were able to differentiate between two types of SH, non-soliciting and soliciting SH behaviours, and to examine their association with mental well-being. Non-soliciting SH can be regarded as an indicator of a more general climate of SH at the workplace while soliciting SH can be regarded as representing experiences of more explicit attempts to create a sexual relation between two individuals [29]. Our study results indicate that men are affected primarily by non-soliciting SH but possibly less, compared to women, by soliciting SH. A similar distinction between different forms of SH is found in other research differentiating between ambient SH, representing a hostile environment, and direct SH, representing a more direct sexual approach [35]. In a survey of university staff by Berdahl et al a striking gender difference was seen regarding men’s and women’s evaluation of ambient and direct SH experiences, where a vast majority of the women perceived direct SH negatively while the reverse was true for men [35]. Ambient SH experiences, on the other hand, were perceived equally negative by both genders in the same study. This agrees with the gender differences found in our result concerning unwelcome SH experiences. In our study, men primarily reported experiences of non-soliciting SH, which was also the only type of SH that was significantly associated with any of the outcomes within this group, specifically with LV. Among women, on the other hand, experiences of non-soliciting SH were related to both PMH and LV when in combination with experiences of soliciting SH. That women evaluate the same SH experience more threatening than men has been explained by gender differences in power generating unequal control over the situation and options in responses [35,36]. This could partly explain why men do not experience direct SH as threatening as women, and possibly are less negatively affected by it, in terms of mental well-being outcomes, compared to women. These findings underscore the complexity of SH experiences and their possible varied impact based on gender. However, our result regarding gender differences between type of SH exposure and mental well-being should be interpreted with caution as relatively few men reported experiences of SH in combination with poor mental well-being outcomes. More research examining the consequences of different types of SH in relation to gender is needed to further clarify possible gender differences.

Also, regarding gender differences in outcomes related to SH, the interaction analyses result did not support our hypothesis that women would be more vulnerable regarding the development of poor mental well-being after exposure to SH, compared to men. However, the analysis of the association between SH and mental well-being, when performed separately by gender, showed a statistically significant association only for one outcome (LV) among men, while among women a statistically significant association was found for both outcomes (LV and PMH), supporting our hypothesis of a gender difference regarding the impact of SH on mental well-being. Also, the reported level of SH exposure during the last three years was considerably higher among women (14%) compared to men (5%) which might suggest overall more negative consequences of SH for women compared to men.

Further, our interaction analyses indicate that younger men, compared to older, might be more vulnerable when it comes to mental well-being consequences of SH. It is possible that younger generations perceive and react to SH differently than older due to differences in perception and awareness of what constitutes SH. However, our result is based on a low number of young men exposed to SH and should be interpreted with caution until it is tested in a larger, prospective study design. Also, we found indications that women with a foreign background might be more vulnerable regarding negative mental-well-being reactions after SH exposure compared to women with a Swedish background. This is in line with what other authors suggest, based on the rationale that minorities might face other stressors on top of SH, such as racism, discrimination, or lack of supporting network interacting with the reactions to SH experiences [11]. Further, to have a ‘low’ academic or other professional position compared to a ‘high’ academic position seems to exacerbate the harm of SH in both women and men. The academic workplace setting has its unique features including a mixture of formal and informal power structures related to, for example, professional rank, teacher–student relations, career success and informal networks. A ‘high’ academic position, being a professor or senior lecturer, generally represents being in a position of higher power in this setting. Our result indicates that employees with less powerful professional positions might be more negatively affected by their SH experience. However, the interaction effect is more prominent among men compared to women. Evidence from other studies shows that SH is more commonly experienced by women in the highest occupational groups [3]. This is coherent with the theory that SH stems from the desire to maintain social status in a gender-based hierarchy [37]. According to this view, men may see women in high-ranking jobs as threats to their own status, motivating SH behaviours. This means that women in ‘high’ academic positions on the one hand have a power position linked to their professional position and on the other hand face an increased risk of SH experiences. Taking this into consideration, it is possible that the gender difference we see in our result reflects a reduced mitigating effect of a ‘high’ academic position among women compared to men. In other words, this suggests that women benefit less from a high academic position compared to men, for example, when exposed to SH experiences.

To our knowledge, our study is the first to examine the relation between SH and vitality, i.e. the individual’s experience of energy and fatigue. Employees experience of vitality has been linked to work-related outcomes, such as creativity and innovative behaviours, valuable traits in academia, where new knowledge and ideas are produced contributing to innovation and societal progress. As our results indicate that SH is a risk factor for LV, it is plausible that efforts to reduce SH in the workplace would increase creativity and innovative behaviours among staff ultimately improving the quality of, for example, research and education in this sector.

## Strengths and limitations of the study

In this study, we used validated instruments to measure mental well-being outcomes, i.e. GHQ-12 for measuring mental health and the vitality scale of the SF-36 instrument for measuring vitality, which is a strength [19,31]. The instrument used to measure SH exposure, the LUSHI instrument, has also been validated in a previous study [29]. Also, by using this instrument we were able to examine two different types of SH behaviour, non-soliciting and soliciting SH, providing new insights regarding different types of SH. The types of SH examined are limited when it comes to effectively distinguishing between various other potentially relevant aspects, such as whether the SH was conducted online or in person, or whether it was physical or non-physical in nature. However, the differentiation between soliciting and non-soliciting SH was based on a factor analysis of the overall SH scale identifying these two factors, supporting the relevance of studying these aspects separately [29]. Another strength of this study is that we were able to control for the potential confounding effect of age, foreign background, professional position and type of employment in the analysis, ensuring more reliable results.

We base our analysis on a cross-sectional survey sent by email to all staff at a large university in Sweden, which included PhD students and administrative staff, with a response rate of 33%. This might be considered a low response rate. However, a comparison between the participants and the target population showed no major differences, supporting the notion that the sample well represented the underlying population [4].

There is a risk for selection bias when using a cross-sectional study design. Individuals whose mental well-being was negatively affected by SH exposure might have left their workplace and thus be underrepresented compared to the ‘underlying cohort’, i.e. a healthy worker effect. Further, the cross-sectional study design entails limitations when it comes to drawing conclusions about the direction of causality. In this study we cannot be sure that exposure to SH preceded mental health outcomes. Although the former was assessed regarding the last three years, the mental health outcomes were assessed at the point in time when the questionnaire was completed and concerned the past few weeks for mental health and past four weeks for vitality, which seem to make it more probable that SH preceded the onset of poor mental health in the majority of cases. Also, previous studies have convincingly established a causality link between SH and poor mental well-being, showing a decrease in mental well-being outcomes after SH exposure, which is why we find it plausible that our study result reflects an impact of SH exposure on mental well-being among our study participants [12–14,26].

Finally, when asked to participate in a survey asking about SH experiences people with relevant experiences may choose not to participate because they do not want to be reminded of uncomfortable events which might lead to an under-representation of those exposed. However, the opposite might also be true, that people without SH experience might think that the survey is not relevant to them.

## Conclusions

Our study result showing that SH is a significant risk factor for poor mental health among female employees and for low vitality among both female and male employees at a large university in Sweden suggests that SH experiences within academia require attention and action. Women reported higher levels of SH compared to men, which indicates an overall more negative impact of SH on mental well-being among women compared to men. Considering that men are overall less affected by SH at the workplace, there is a risk that male employees, including men in leading positions, underestimate the magnitude of the problem. Therefore, we believe that equal representation of genders at the management level and other power positions is crucial to counteract SH at workplaces. Although men are less affected compared to women, our study indicates that SH still has an impact on male employees. Thus, countermeasures should be inclusive and directed at all employees, regardless of gender, to foster a safer and more supportive work environment for everyone.

## Data Availability

Data cannot be shared publicly because of its sensitive nature. Data are available from Lund University (contact via the correspondent author) for researchers who meet the criteria for access to confidential data.
